# The geographic distribution of priority population groups for the elimination of mother-to-child transmission of HIV in South Africa

**DOI:** 10.1371/journal.pone.0231228

**Published:** 2020-04-08

**Authors:** Faith Moyo, Ahmad Haeri Mazanderani, Tendesayi Kufa, Gayle G. Sherman

**Affiliations:** 1 Centre for HIV & Sexually Transmitted Infections, National Institute for Communicable Diseases, National Health Laboratory Service, Johannesburg, South Africa; 2 School of Public Health, Faculty of Health Sciences, University of the Witwatersrand, Johannesburg, South Africa; 3 Paediatric HIV Diagnostics Division, Wits Health Consortium, Johannesburg, South Africa; 4 Department of Medical Virology, Faculty of Health Sciences, University of Pretoria, Pretoria, South Africa; 5 Department of Paediatric and Child Health, Faculty of Health Sciences, University of the Witwatersrand, Johannesburg, South Africa; Ohio State University, UNITED STATES

## Abstract

**Background:**

Women of reproductive age living with HIV (WRLHIV), HIV-positive pregnant women, adolescent girls and young women (AGYW) are key populations for eliminating mother-to-child of HIV (eMTCT) in South Africa. We describe the geographical distribution of WRLHIV, their pregnant counterparts and AGYW for risk-adjusted allocation of eMTCT interventions.

**Methods:**

For the year 2018, we triangulated data from the Thembisa Model with five routine HIV-related and demographic data sources to determine the distribution of WRLHIV (15–49 years) and AGYW (15–24 years) nationally and by province. Data analysed included total population estimates, number of live-births, live-births to HIV-positive women, age-specific HIV prevalence rates, intrauterine (IU)-transmission rates and IU-case rates/100 000 live-births. IU-transmission rates and IU-case rates were calculated from de-duplicated routine HIV test-data for neonates (aged <7days). Data de-duplication was achieved by a patient-linking algorithm that uses probabilistic matching of demographics (name, surname, date of birth), supplemented by manual matching to account for spelling errors.

**Results:**

There were 58 million people in South Africa in 2018. Females (all ages) constituted 51% of the population. Women of reproductive age constituted 27% and AGYW constituted 8% of the total population. WRLHIV, AGYW living with HIV and HIV-positive pregnant women accounted for 7%, 0.8% and 0.4% of the total population respectively. Gauteng was the most populous province followed by KwaZulu-Natal, with Western Cape and Eastern Cape in third and fourth positions. The distribution of WRLHIV and AGYW followed a similar trend. However, Mpumalanga and Limpopo provinces had higher proportions of WRLHIV and AGYW living with HIV ahead of Western Cape. KwaZulu-Natal had the highest number of live-births to HIV-positive women. The national IU-transmission rate of <1% translated into 241 cases/100 000. While provincial IU-case rates were fairly similar at 179–325, districts IU-case rates varied, ranging from 87–415 cases/100 000 live-births.

**Conclusion:**

Findings suggest that the need for eMTCT interventions is greatest in Gauteng, KwaZulu-Natal, Western Cape and Eastern Cape. Limpopo and Mpumalanga provinces may require more HIV prevention and family planning services because of high fertility rates, high number of WRLHIV and AGYW living with HIV. eMTCT will require robust viral load monitoring among WRLHIV, pregnant and breastfeeding women. The national laboratory database can provide this service near-real time.

## Introduction

Women constitute the highest proportion of people living with HIV globally [1]. In South Africa, the HIV epidemic also disproportionately affects women. In 2017, approximately two thirds of people living with HIV, aged 15–49 years and on antiretroviral therapy (ART), were female [2]. In the same year, disparities in HIV prevalence by sex were most pronounced among young adults. HIV prevalence among 20–24 year olds was three times higher in females (15.6%) compared to males (4.8%) [2]. Potential reasons for gender disparities in the profile of the HIV epidemic are both economic and sociocultural [3]. Extensive geospatial heterogeneity in the distribution of the HIV epidemic has been reported previously [4]. In 2018, provincial HIV prevalence estimates among people living with HIV aged 15–49 years ranged from 6.7% in the Western Cape to 18.4% in KwaZulu-Natal [4]. While poorly understood, variations in provincial HIV prevalence rates have been linked to differences in the uptake of HIV prevention interventions and societal norms between provinces [4]. Thus, provincial variations in the profile of the HIV epidemic necessitate optimized allocation of resources and targeted interventions for HIV/AIDS programmes. This is particularly pertinent for the prevention of mother-to-child transmission of HIV (PMTCT) programme, which has prioritized the elimination of mother-to-child transmission of HIV (eMTCT). The national MTCT rate of 1.0% at birth in 2017 equalled just under 250 cases per 100 000 live births [5]. However, this figure was five times higher than the World Health Organization (WHO) eMTCT target, defined as ≤50 cases per 100 000 live births at completion of breastfeeding and an overall mother-to-child transmission (MTCT) rate of <5% in breastfeeding populations [5]. Sub-national level variations in MTCT rates also exist, with some districts reporting almost double the national MTCT rate at birth [6]. This suggests the need for contextualized and tailored responses to maternal and paediatric HIV in South Africa.

We describe the distribution of women of reproductive age (15–49 years) living with HIV (WLHIV) as well as those without HIV by province in South Africa, in order to identify areas that may require additional eMTCT interventions. These may include interventions aimed at reducing the risk of HIV acquisition by women of reproductive age and unplanned pregnancies among WRLHIV (pillars 1–2 of the South African PMTCT strategy) [7]. Women of reproductive age living with HIV and sub-groups (adolescent girls and young women (AGYW) aged 15–24 years) are priority populations for achieving eMTCT. These women can potentially fall pregnant and transmit HIV to their infants. While the risk of vertical transmission of HIV is low in planned pregnancies, where WRLHIV fully access and utilize preconception and post conception PMTCT services, the contrary is true for unplanned pregnancies [8]. Pregnancies are more likely to be unplanned in AGYW compared to older women [9]. In addition, AGYW represent a sub-population that will remain in the reproductive age group for the longest time; are associated with high incident infections and where HIV positive, AGYW have poorer PMTCT outcomes compared to older WRLHIV [9]. Therefore, the risk of MTCT is higher in this population. However, an increase in number of women conceiving on ART is anticipated with the scale up of the universal test and treat strategy [10]. Consequently, understanding where WRLHIV, including AGYW (regardless of their HIV status) and those who are pregnant, are located in South Africa is key for eMTCT. Scaling up family planning services and ART/PMTCT services (for example, stock planning of Efavirenz based regimens and Dolutegravir rollout in relation to new ART guidelines) in geographic areas with a high burden of these groups may be beneficial for maternal HIV care and eMTCT.

Obtaining accurate estimates of vertical transmission is challenging in South Africa because of the lack of a unique patient identifier in the health system. Monitoring of early infant MTCT rates and coverage of early infant diagnosis currently relies on routine data from the District Health Information System (DHIS) and the National Health Laboratory Service (NHLS) [11]. There are no accurate, national data sources for postnatal transmission rates, breastfeeding practices, maternal virological control during pregnancy and the postpartum period. An analysis of current data sources used for tracking eMTCT, their strengths and weakness, are described elsewhere by Sherman et al [11]. Using six different data sources, we describe the distribution of WRLHIV in South Africa during 2018 in relation to where the intra-uterine transmissions occur.

## Materials and methods

Data for these analyses were extracted from six independent data sources for HIV-related and demographic indicators from the public health sector in South Africa. These were the Thembisa Model, DHIS, Statistics South Africa (Stats SA), the South African National HIV Prevalence, Incidence, Behaviour and Communication Survey (SABSSM), the National Antenatal Sentinel HIV & Syphilis Survey Report, (herein referred to as the ANC sero-prevalence survey) and the National Institute for Communicable Diseases’ Surveillance Data Warehouse (NICD SDW), a division of the NHLS. The Thembisa Model was used as the primary data source because it had most of the indicators required for the analyses. The other sources were used for triangulation purposes where applicable. The reporting period was the year 2018. Where data for 2018 was not available, available data from the most recent year were used. A brief description of each data source and indicators that were used for the analyses follows (Table 1).

**Table 1:** Indicators used in the analyses by data sources.

Indicators	Data Sources					
	Thembisa Model	Stats SA	DHIS	ANC sero-prevalence survey	SABSSM	NICD SDW
*Total Population estimates (males and females*, *all ages)*	✔	✔				
*Total Population for females (All ages)*	✔	✔				
*Total Population for females 15–49 years*	✔	✔				
*Total Population for females 15–24 years*	✔	✔				
*Number of live births*	✔	✔	✔			
*Total births to HIV-positive women*	✔		✔			
*HIV prevalence in females 15–49 years*	✔					
*HIV prevalence in females 15–24 years*	✔					
*HIV prevalence in pregnant females 15–49 years*				✔		
*HIV prevalence in pregnant females*	✔					
*HIV prevalence in adults 15–49 years*	✔	✔			✔	
*HIV PCR tests at age <7 days (Birth HIV PCR)*						✔
*Total fertility rate*	✔	✔				
**Reporting Unit**	National Province	National Province	National Province District Sub-district Facility	National Province District	National*	National Province District Sub-district Facility
**Year for which data was available**	2018	2018	2018	2017	2017	2018

**Abbreviations**: Stats SA Statistics South Africa; DHIS Demographic Health Information System; PCR polymerase chain reaction; ANC antenatal care; NICD National Institute for Communicable Diseases SDW Surveillance Data Warehouse; SABSSM South African National HIV Prevalence, Incidence, Behaviour and Communication Survey.

*Data available at national level only at time of publication. Blocked out areas indicate absence of data for a specific indicator per data source.

### 1. Description of data sources

#### a) Thembisa Model

The Thembisa Model is an integrated epidemiological and demographic mathematical model developed to describe the South African HIV epidemic and to evaluate the impact of HIV prevention and treatment strategies [12]. The model also provides demographic statistics and is used to evaluate the demographic impact of HIV in South Africa [12]. The model provides data at national and provincial level. Data were obtained from Thembisa 4.1, published in 2018.

#### b) DHIS

The DHIS collects aggregated data on health services provided by all health facilities in the public sector in South Africa since 2001. The data are collected from paper-based registers at facility level then entered electronically onto the DHIS software for collation at sub-district, district, provincial and national level on a monthly basis [13]. In some facilities, the data is collected from Tier.net, a national electronic HIV register for aggregation onto DHIS.

#### c) STATS SA statistical release P0302 and P0305

The P0302 document provides data on mid-year population estimates for South Africa and the nine provinces on an annual basis. The projections are based on the cohort component method for population estimation which has been described elsewhere [14]. The population estimates approximate the actual population as at 1 July of a given year. The P0305 document provides data on recorded live births in the public sector in South Africa. The data are based on births registered in the national birth registry system, which is collated and maintained by the Department of Home Affairs in South Africa. The latest P0302 and P0305 documents were released in 2018 [14–15] at the time of writing.

#### d) National antenatal sentinel HIV & syphilis survey report (ANC sero-prevalence survey)

South Africa has been conducting the ANC sero-prevalence survey annually to monitor HIV & syphilis prevalence among pregnant women aged 15–49 years, attending antenatal clinics since the 1990s [16]. The survey is cross sectional by design and is conducted across the 52 health districts in South Africa. Sentinel sites are randomly selected from public sector facilities across the country using probability proportional to size sampling methods [10]. The most recent report for the 2017 survey was published in 2019 [10].

#### e) SABSSM survey

The SABSSM is a population based, cross sectional survey of all households in South Africa that evaluates trends in HIV prevalence and other health indicators related to HIV across the country. The surveys are conducted every five years. At the time of writing, only an executive summary of findings from the latest 2017 survey was available [2].

#### f) National Institute for Communicable Diseases (NICD) Surveillance Data Warehouse (SDW)

All pathology tests performed in the public sector are processed by the National Health Laboratory Service through a network of about 260 laboratories across South Africa [17]. A single Laboratory Information System (LIS) used by all laboratories stores data pertaining to test specimens (viz. patient identifiers, clinical and geographic information). These data are archived in near real-time in a central data repository called the Surveillance Data Warehouse (SDW) of the NICD within the NHLS.

### 2. Data management and analysis

The distribution of WRLHIV and AGYW for the year 2018 was described with respect to the following:

#### a) Total population estimation

Total population estimates were extracted from the Thembisa Model and Stats SA to describe: i) overall population ii) total population of women of all ages and ii) total population of WRLHIV (15–49 years) and iii) total population of AGYW (15–24 years) and iv) total population of AGYW living with HIV nationally and by province.

#### b) Number of live births

The number of births (Thembisa Model), number of live births at a facility (DHIS) and number of registered live births from Stats SA were extracted nationally and by province and used to describe child-birth patterns by geographic location.

#### c) Number of WRLHIV

In order to estimate the number of WRLHIV in South Africa, the total population estimates for women aged 15–49 years were multiplied by the mean HIV prevalence rate amongst women aged 15–49 years. To estimate number of pregnant WRLHIV, total births to HIV-positive women were extracted from the Thembisa Model. For comparison, number of live births from Stats SA were multiplied by the HIV prevalence rate amongst pregnant women aged 15–49 years obtained from the ANC sero-prevalence survey.

#### d) Number of HIV-positive AGYW

The number of AGYW living with HIV were calculated by multiplying the total population of AGYW by the prevalence of HIV in females aged 15–24 years obtained from the Thembisa Model.

#### e) Number of live births to women living with HIV

The indicators used to provide these data were obtained from the Thembisa Model and compared to i) number of live births to women living with HIV (DHIS), number of HIV-exposed infants (obtained by multiplying the number of registered live births from Stats SA by the HIV prevalence estimates from the ANC sero-prevalence survey) and iii) number of HIV PCR tests performed among neonates aged <7 days from the NICD SDW, considering that birth testing coverage for HIV-exposed neonates is >95%. These data were also used as a proxy for number of HIV-positive, pregnant women as explained in 2c.

#### f) Intra-uterine transmission rates

These were calculated as the number of de-duplicated HIV PCR positive tests performed at birth (<7 days of life) divided by total HIV PCR tests performed at birth expressed as a percentage from the NICD SDW. The number of intra-uterine infections per 100 000 live births was also reported (intra-uterine case rates). Test data for HIV PCR positive neonates were de-duplicated by a patient-linking algorithm that uses probabilistic matching of demographics (for example name, surname, date of birth) supplemented by manual matching to account for spelling errors. Because of availability of data, intra-uterine transmission rates were also calculated to district level. Intra-partum and postnatal case rates could not be accurately calculated from the SDW because of the lack of a unique identifier to allow for longitudinal monitoring.

### Ethics clearance

Part of this work utilized routinely collected surveillance data for HIV programs under ethical clearance issued to the NICD (M160 667) by the Human Research Ethics Committee (HREC) of the University of the Witwatersrand. This clearance waives the requirement for patient consent for studies conducted by the NICD, which audit routine programmatic data from the national HIV surveillance programme. Patient identity was protected by anonymizing the data prior to analysis.

## Results

**Fig 1:**
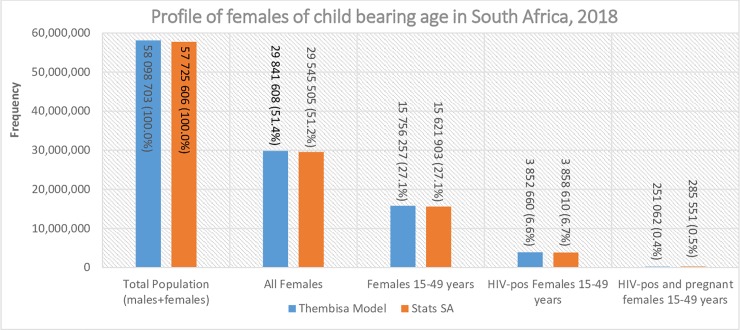
Profile of females of reproductive age in South Africa in 2018. **Abbreviation**: Stats SA Statistics South Africa.

**Fig 2:**
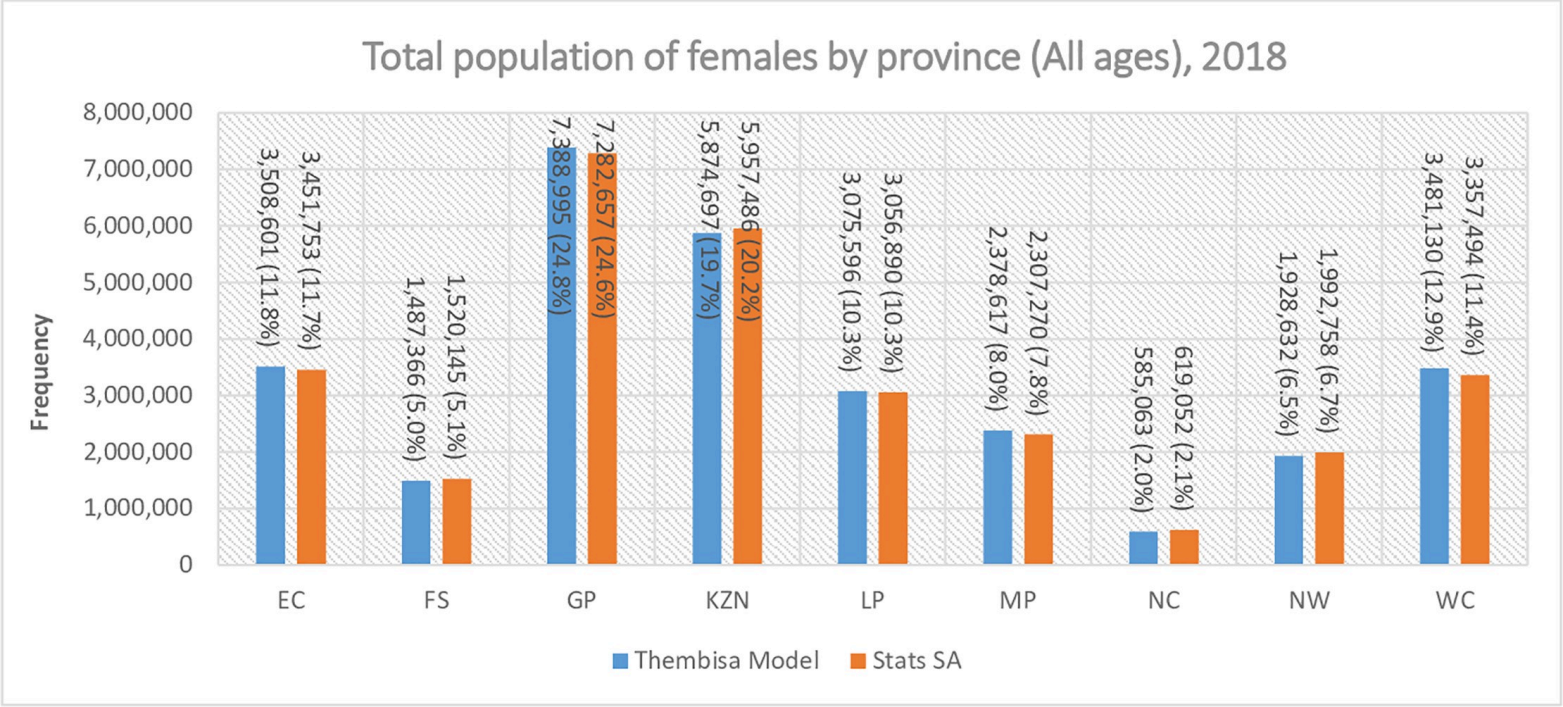
Population density of females by province in South Africa, 2018. **Abbreviations**: EC Eastern Cape, FS Free State, GP Gauteng Province, KZN KwaZulu-Natal, LP Limpopo Province, NC Northern Cape, NW North West, WC Western Cape, Stats SA Statistics South Africa.

**Fig 3:**
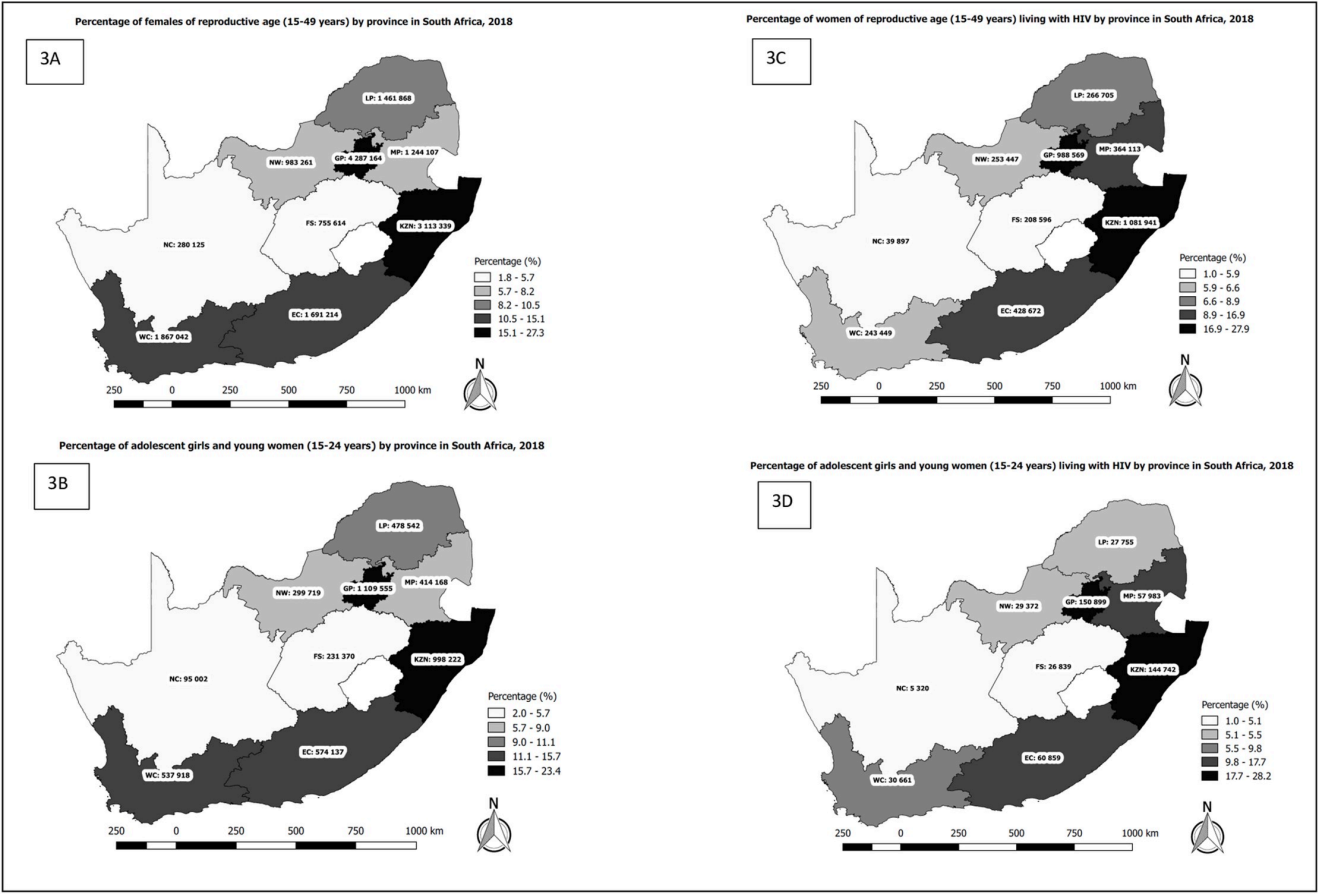
**Provincial distribution of percentages of**: 3A) females of reproductive age 3B) adolescent girls and young women 3C) women of reproductive age living with HIV 3D) adolescent girls and young women living with HIV in South Africa, 2018. **Source**: Thembisa Model 4.1. **Abbreviations**: EC Eastern Cape, FS Free State, GP Gauteng Province, KZN KwaZulu-Natal, LP Limpopo Province, NC Northern Cape, NW North West, WC Western Cape.

There were approximately 58 million people in South Africa in 2018 with half of the population (51%) being female (Fig 1). Women of reproductive age constituted 27% of the total population, while WRLHIV (3.8 million) accounted for approximately 7% of the total population. There was a total of 4.7 million (8.1%) AGYW of which 477 798 (10.1%) were living with HIV in South Africa. Gauteng province had the highest number of females (all ages) followed by KwaZulu-Natal (KZN), the Eastern Cape and the Western Cape (Fig 2). The Northern Cape had the lowest number of females during the analysis period. A similar trend was observed for women of reproductive age (Fig 3A) and AGYW (Fig 3B), where 69.5% and 67.9% occurred in the four most populous provinces, respectively. However, the analysis of the geographic distribution of WRLHIV (Fig 3C) and HIV-infected AGYW (Fig 3D) showed that 74.4% and 86.7% respectively, of these women were located in four provinces. The latter comprised three of the four most populous provinces but included Mpumalanga instead of the Western Cape. KwaZulu-Natal and Gauteng provinces recorded the highest numbers of WRLHIV at approximately 1 million each, contributing 53.7% of the total population of WRLHIV in the country followed by the Eastern Cape, Mpumalanga and Limpopo provinces.

Overall, out of approximately 1 million live births in the country; between 250 000–286 000 births occurred to women living with HIV in 2018 (Table 2). Limpopo province had the third highest number of live births in 2018 ahead of the Eastern Cape and Western Cape provinces (Table 2). This was in keeping with Limpopo province having the highest fertility rate throughout the country at (3.1%) (Table 2). KwaZulu-Natal province recorded the highest number of live births to women living with

**Table 2:** Number of live births (total), number of live births to women living with HIV and intra-uterine transmission rates by province in South Africa, 2018.

Province	Total Population	Number of Live births (Total)			Live births to women living with HIV				Fertility rates		HIV PCR positive (<7 days)	IU transmission rate	IU Case rate/100 000
	Thembisa Model	Thembisa Model	Stats SA	DHIS	Thembisa Model	Stats SA	DHIS	NICD SDW	(Stats SA)	Thembisa Model	NICD SDW		
EC	6 593 566 (11.3%)	126 530 (10.8%)	105 796 (11.4%)	104 016 (11.0%)	27 448 (10.9%)	31 950 (11.9%)	32 164 (11.2%)	31 316 (11.7%)	2,8%	2,5%	248	0,8%	238
FS	2 875 955 (5.0%)	57 744 (5.0%)	47 306 (5.1%)	47 062 (5.0%)	13 553 (5.4%)	14 097 (5.3%)	14 386 (4.9%)	14 963 (5.6%)	2,6%	2,5%	106	0,7%	225
GP	14 931 713 (25.7%)	280 597 (24.1%)	205 612 (22.2%)	219 958 (23.2%)	54 976 (21.9%)	62 095 (20.6%)	55 497 (21.7%)	56 340 (21.0%)	1,9%	2,0%	471	0,8%	235
KZN	11 214 103 (19.3%)	231 742 (19.9%)	190 923 (20.6%)	195 292 (20.6%)	70 126 27.9%)	84 770 (29.2%)	78 662 (29.7%)	75 195 (28.1%)	2,6%	2,3%	453	0,6%	254
LP	5 794 578 (10.0%)	140 604 (12.1%)	123 414 (13.3%)	122 932 (13.0%)	19 885 (7.9%)	26 781 (9.4%)	25 265 (9.4%)	24 978 (9.3%)	3,1%	3,1%	264	1,1%	227
MP	4 650 305 (8.0%)	94 957 (8.1%)	77 353 (8.3%)	79 669 (8.4%)	24 708 (9.8%)	26 996 (9.8%)	26 558 (9.5%)	26 350 (9.8%)	2,7%	2,4%	245	0,9%	325
NW	3 914 669 (6.7%)	76 700 (6.6%)	55 094 (5.9%)	58 097 (6.1%)	17 119 (6.8%)	16 087 (5.8%)	15 746 (5.6%)	16 353 (6.1%)	2,7%	2,9%	157	1,0%	283
NC	1 154 340 (2.0%)	25 451 (2.2%)	24 195 (2.6%)	21 549 (2.3%)	3 024 (1.2%)	4 597 (1.5%)	3 970 (1.6%)	4 192 (1.6%)	2,6%	2,5%	42	1,0%	179
WC	6 809 913 (11.7%)	122 076 (10.5%)	97 298 (10.5%)	98 535 (10.4%)	14 389 (5.7%)	18 389 (6.5%)	17 395 (6.4%)	18 198 (6.8%)	2,0%	2,1%	176	1,0%	183
**RSA**	**58 098 703**	**1 166 440**	**927 113**	**947 110**	**251 062**	**285 551**	**269 643**	**267 887**	**2,4%**	**2,4%**	**2 162**	**0,8%**	**241**

**Abbreviations**: EC Eastern Cape; FS Free State; GP Gauteng Province; KZN KwaZulu-Natal; LP Limpopo Province; MP Mpumalanga Province; NC Northern Cape; NW North West; WC Western Cape; RSA South Africa; Stats SA Statistics South Africa; DHIS District Health Information System; NICD National Institute for Communicable Diseases’ Surveillance Data Warehouse

HIV, followed by Gauteng and the Eastern Cape respectively (Table 2). Similarly, KZN had the highest HIV prevalence rate among pregnant women (S1 Table) followed by Mpumalanga province (S1 Table). The intra-uterine transmission rate for South Africa was 0.8%, translating into 241 cases of HIV-infected neonates per 100 000 live births (Table 2). Provincial intra-uterine transmission rates ranged from 0.6% in KZN to 1.1% in Limpopo province. Districts intra-uterine transmission rates ranged from 0.4% to 1.7% with KZN districts recording intra-uterine transmission rates lower than the national average of 0.8% (Table 3). Provincial intra-uterine case rates were fairly similar and ranged from 179–325 cases per 100 000 live births in the Northen Cape and Mpumalanga respectively. District level intra-uterine case rates had a greater spread, ranging from 87–415 cases per 100 00 live births (Table 3). Overall, the four provinces with the most WRLHIV yielded 1 417 (65.5%) of all intra-uterine infections in 2018. In addition, Limpopo’s intra-uterine infections contributed a further 264 (12.2%). Therefore focussing eMTCT efforts in five provinces has the potential to reduce 1 681 (77.8%) of all intra-uterine infections per annum, nationally.

**Table 3:** Intra uterine transmission rates and intra uterine case rates per 100 000 live births by geographic location in South Africa, 2018.

Geographic Area	District	ANC prevalence among pregnant women*(ANC seroprevalence survey)	Registered live births per year (Stats SA)	Total PCR <7 days	PCR Positive (<7 days)	IU transmission rate	IU Case rate/100 000
**South Africa**		**30,8%**	**897750**	**267 887**	**2 162**	**0,8%**	**241**
Eastern Cape	**Total**	**30,2%**	**104325**	**31 316**	**248**	**0,8%**	**238**
Eastern Cape	Alfred Nzo	26,6%	18230	4 171	28	0,7%	154
	Amathole	28,3%	11070	3 092	23	0,7%	208
	Buffalo City Metro	31,2%	17468	4 156	30	0,7%	172
	Chris Hani	31,9%	11178	3 839	22	0,6%	197
	Joe Gqabi	28,3%	2475	1 476	9	0,6%	364
	Nelson Mandela Bay Metro	29,9%	16717	3 717	54	1,5%	323
	O R Tambo	33,3%	21847	9 068	63	0,7%	288
	Sarah Baartman	25,4%	5340	1 797	19	1,1%	356
Free State	**Total**	**29,8%**	**47047**	**14 963**	**106**	**0,7%**	**225**
	Fezile Dabi	26,5%	7353	2 570	15	0,6%	204
	Lejweleputswa	27,3%	9832	3 260	21	0,6%	214
	Mangaung Metro	31,7%	15559	4 475	29	0,6%	186
	Thabo Mofutsanyana	31,0%	12942	4 328	38	0,9%	294
	Xhariep	35,1%	1361	330	3	0,9%	220
Gauteng	**Total**	**30,2%**	**200726**	**56 340**	**471**	**0,8%**	**235**
	City of Johannesburg Metro	29,6%	57855	17 317	165	1,0%	285
	City of Tshwane Metro	25,3%	62923	12 524	109	0,9%	173
	Ekurhuleni Metro	31,6%	57406	17 761	152	0,9%	265
	Sedibeng	34,0%	14835	4 039	17	0,4%	115
	West Rand	35,5%	7707	4 699	28	0,6%	363
KwaZulu-Natal	**Total**	**44,4%**	**178456**	**75 195**	**453**	**0,6%**	**254**
	Amajuba	39,7%	8199	3 303	22	0,7%	268
	eThekwini Metro	46,2%	55992	23 409	139	0,6%	248
	Harry Gwala	39,5%	8371	3 050	18	0,6%	215
	iLembe	44,3%	9170	4 616	27	0,6%	294
	Ugu	45,9%	12595	5 659	31	0,5%	246
	uMgungundlovu	46,2%	14523	7 173	47	0,7%	324
	uMkhanyakude	46,3%	13232	6 067	26	0,4%	196
	Umzinyathi	36,7%	11867	3 640	21	0,6%	177
	uThukela	36,3%	11441	4 577	17	0,4%	149
	uThungulu	45,9%	16894	7 403	55	0,7%	326
	Zululand	48,4%	16172	6 298	50	0,8%	309
Limpopo	**Total**	**21,7%**	**116276**	**24 979**	**264**	**1,1%**	**227**
	Capricorn	21,6%	26133	5 847	61	1,0%	233
	Greater Sekhukhune	22,6%	25747	5 098	50	1,0%	194
	Mopani	24,5%	22170	5 335	53	1,0%	239
	Vhembe	16,8%	30009	4 560	54	1,2%	180
	Waterberg	25,8%	12217	4 139	46	1,1%	377
Mpumalanga	**Total**	**34,9%**	**75369**	**26 350**	**245**	**0,9%**	**325**
	Ehlanzeni	38,5%	39407	13 524	128	0,9%	325
	Gert Sibande	38,6%	15886	7 195	66	0,9%	415
	Nkangala	25,1%	20076	5 631	51	0,9%	254
North West	**Total**	**29,2%**	**55392**	**16 353**	**157**	**1,0%**	**283**
	Bojanala Platinum	33,8%	18421	6 299	54	0,9%	293
	Dr Kenneth Kaunda	30,9%	12291	3 618	37	1,0%	301
	Dr Ruth Segomotsi Mompati	22,1%	9182	2 314	24	1,0%	261
	Ngaka Modiri Molema	23,9%	15498	4 122	42	1,0%	271
Northern Cape	**Total**	**19,0%**	**23402**	**4 192**	**42**	**1,0%**	**179**
	Frances Baard	24,3%	8957	1 733	15	0,9%	167
	John Taolo Gaetsewe	21,9%	5162	1 171	7	0,6%	136
	Namakwa	2,9%	1550	113	2	1,8%	129
	Pixley Ka Seme	15,8%	2843	540	7	1,3%	246
	ZF Mgcawu	14,5%	4890	635	11	1,7%	225
Western Cape	**Total**	**18,9%**	**95997**	**18 199**	**176**	**1,0%**	**183**
	Cape Winelands	15,2%	13354	2 127	25	1,2%	187
	Central Karoo	11,8%	1156	109	1	0,9%	87
	City of Cape Town Metro	21,6%	63800	13 288	121	0,9%	190
	Eden	15,7%	9062	1 415	18	1,3%	199
	Overberg	19,8%	3855	723	6	0,8%	156
	West Coast	13,8%	4770	537	5	0,9%	105

**Abbreviations**: Stats SA Statistics South Africa; PCR polymerase chain reaction; ANC antenatal care; IU intra-uterine.

*ANC sero-prevalence survey was used because the Thembisa Model does not provide district level estimates.

## Discussion

In 2018, South Africa’s population of 58 million people was evenly distributed between males and females. Gauteng province had the highest number of females, accommodating a quarter of the total population of females (all ages). The majority of all women, 20 253 423 (68%), and women of reproductive age, 10 958 758 (70%), were located in the densely populated provinces; that is Gauteng, KwaZulu-Natal, Western Cape and Eastern Cape provinces (“the big four”). Although the distribution of WRLHIV followed a similar trend, Mpumalanga and Limpopo had the fourth [364 113 (10%)] and fifth, [266 705 (7%)] highest number of WRLHIV ahead of the Western Cape. Adolescents and young women were also mostly located in the big four provinces, 3 219 832 (68%). Ten percent (477 798) of all AGYW were living with HIV in South Africa in 2018. Gauteng, KwaZulu-Natal, Eastern Cape and Mpumalanga, had the highest percentage of AGYW living with HIV at 32%, 30%, 13% and 12% respectively. Although the national intra-uterine transmission rate was <1.0%, 241 per 100 000 neonates acquired HIV from their mothers in 2018 at birth. Mpumalanga, North West and KwaZulu-Natal had intra-uterine case rates that were above the national average at 325, 283 and 254 cases per 100 000 live births. Districts intra-uterine case rates varied greatly and ranged from as low as 87–415 cases per 100 000 live births. However, even districts with the lowest intra-uterine transmission rate at birth, still had case rates higher than the eMTCT target without taking infections after birth into account.

Based on the demographic profiles of provinces, findings of these analyses suggest disparities in the need for eMTCT interventions. In this last mile to eMTCT, it may be worthwhile strengthening interventions in provinces that have the largest numbers of WRLHIV (north eastern parts of the country) as well as those regions with the highest intra-uterine transmission rates and intra-uterine case rates while maintaining current levels of support elsewhere. Although early transmission rates are below the eMTCT target of <5%, South Africa is not positioned to achieve eMTCT case rate targets because of the extremely high maternal HIV seroprevalence [18]. However, the country has a well-established PMTCT programme in place. Closing PMTCT gaps [5] and scaling up sexual and reproductive health for preventing vertical transmission of HIV could represent low hanging fruit. Sexual and reproductive health services should include intensifying family planning and contraception services amongst all women of reproductive age regardless of HIV status and providing pre-exposure prophylaxis to AGYW. As expected, most women & AGYW were situated in the ‘big four’ provinces. However, we found that the burden of HIV in women and AGYW was also pronounced in Limpopo and Mpumalanga provinces. Limpopo province has the highest fertility rate in the country and a high proportion of AGYW without HIV who may benefit from family planning and pre-exposure prophylaxis initiatives. Mpumalanga also has a significant amount of WRLHIV who may be in need of family planning services. Cross border mobility of pregnant women seeking healthcare from neighbouring countries may be a contributing factor to high WRLHIV and fertility rates in these two provinces.

The findings from our analysis of intra-uterine transmission rates did not necessarily follow the underlying population densities. We observed lower transmission rates in KwaZulu-Natal which has the highest number of WRLHIV and live births to women living with HIV. We attribute the low intra-uterine rates in this province and its districts to the larger denominator (total live births, Table 2) and/or a more effective PMTCT programme. The converse probably explains the high intra-uterine transmission rate in the Northern Cape and its districts, where smaller denominators result in higher intra-uterine rates. Poor de-duplication of HIV PCR positive test data may also result in some provinces like the Western Cape having intra-uterine transmission rates that are above the national average. These results suggest that case rates per 100 000 may be a better indicator for monitoring programme performance at the various geographic levels [18] as it is directly linked to the performance of the PMTCT programme, for example coverage of services and attainment of viral suppression in pregnancy. Since most provincial level intra-uterine case rates were fairly similar, it is necessary to tackle the intra-uterine case rates at district level where there is considerable variation. Districts with high intra-uterine case rates deserve special attention with respect to improving PMTCT services.

Despite the distinct geographical differences in WRLHIV and AGYW, attainment of eMTCT will require more than understanding the geographic distribution of all WRLHIV. In order to fast-track eMTCT, it is critical to target PMTCT pillars of prevention of HIV infection [7] and family planning particularly in AGYW. The proposed School Health Programme which caters for sexual and reproductive health education and HIV testing in schools among children aged ≥12 years, has the potential to reduce maternal HIV prevalence in future and enable eMTCT. Among AGYW with HIV and other WRLHIV, the focus needs to shift towards virologic control, with specific attention to pregnant women, especially considering maternal viral load is the strongest predictor of MTCT risk [19]. Therefore, improving viral load monitoring during pregnancy, delivery and breastfeeding, and improving quality of patient management among WRLHIV and all HIV positive pregnant women may translate into improved viral load suppression rates. Achieving this in the big four provinces may fast-track eMTCT. There is urgent need to effectively track and monitor viral loads of all HIV positive pregnant women. A marker of pregnancy, delivery and the postpartum period within the NICD SDW as recommended in the 2019 National PMTCT guidelines, should allow for monitoring of viral load suppression rates at facility level in near real time to advance UNAIDS 90-90-90 [20] targets in women of reproductive age, particularly in high-risk areas. Such monitoring will provide information on the true picture of viral load burden during pregnancy and prompt rapid intervention. Currently this information is lacking.

In our analyses, the Thembisa Model was used as our primary data source because the model was specifically designed and is used for estimating HIV populations in SA. As a result it contained data for 10/12 indicators reviewed for these analyses. However, estimates may be inaccurate if underlying assumptions are incorrect. We therefore used other data sources to validate the data. Although there were differences in absolute numbers; we found that data from the other sources was generally within the upper and lower limits of the Thembisa model estimates (S2 Table). This may be because the Thembisa Model uses some of the data we triangulated with it, for example Statistics South Africa. Where there were significant differences, we attribute these to differences in (i) methodology or (ii) in reporting intervals or (iii) data quality issues for some of the data sources used for triangulation purposes. For example, the latest data for the ANC seroprevalence survey is 2017, while the reporting period for these analyses was 2018 (S1 Table). The laboratory based data lacks unique patient identifiers therefore de-duplication may be incomplete, resulting in over-estimation of transmission rates. Intra-partum transmission rates would be anticipated to be similar to intra-uterine transmission rates however; postnatal transmissions would depend on breastfeeding practices (e.g. duration of breastfeeding), postnatal virologic control and incident maternal infections which we were not able to measure.

## Conclusions

We have attempted to quantify the need for eMTCT interventions based on the demographic profile of provinces in South Africa. Our results confirm what is mostly known. The need for HIV/eMTCT resources is greatest in Gauteng, KwaZulu-Natal and Eastern Cape. Limpopo and Mpumalanga provinces also warrant attention. While advocating for allocation of interventions for eMTCT on a needs-basis; it is important not to neglect those provinces with the least need. Routine monitoring and surveillance needs to continue until the country reaches eMTCT. Ending paediatric HIV will require robust viral load monitoring & intervention among WRLHIV in general, as well as pregnant and breastfeeding women. The NHLS laboratory data have the potential to provide viral load monitoring service near-real time until clinical databases can assume this role.

## Supporting information

S1 Table HIV prevalence rates by province in South Africa, 2018. (DOCX)

S2 Table 95% Confidence Intervals by indicator: Thembisa Model. (DOCX)
