# Supplementary material for: The geographic distribution of priority population groups for the elimination of mother-to-child transmission of HIV in South Africa
Source: PLoS One. 2020 Apr 8;15(4):e0231228. doi: 10.1371/journal.pone.0231228 (PMC7141689; doi:10.1371/journal.pone.0231228)
Supplement: S1 Table — (DOCX) [file pone.0231228.s001.docx]

**S1 Table.** HIV prevalence rates by province in South Africa, 2018

| **Province** | **HIV prevalence in females 15-49 years (Thembisa Model 2018)** | **HIV prevalence in pregnant females 15-49 years (2017 ANC survey)** | **HIV prevalence in pregnant females (Thembisa Model 2018)** | **HIV prevalence in Adults 15-49 years (males+females, SABSSM 2017)** |
| --- | --- | --- | --- | --- |
| Eastern Cape | 25,4% | 33,7% | 22,5% | 25,2% |
| Free State | 27,7% | 32,7% | 24,2% | 25,5% |
| Gauteng | 23,4% | 32,2% | 20,2% | 17,6% |
| KwaZulu-Natal | 35,1% | 41,1% | 31,4% | 27,0% |
| Limpopo | 18,3% | 23,4% | 15,0% | 17,2% |
| Mpumalanga | 29,7% | 37,3% | 27,0% | 22,8% |
| North West | 26,0% | 27,7% | 22,8% | 22,7% |
| Northern Cape | 14,3% | 17,9% | 12,5% | 13,9% |
| Western Cape | 13,2% | 15,9% | 12,1% | 12,6% |
| **South Africa** | **24,7%** | **30,7%** | **22,1%** | **20,6%** |
