# Supplementary material for: The geographic distribution of priority population groups for the elimination of mother-to-child transmission of HIV in South Africa
Source: PLoS One. 2020 Apr 8;15(4):e0231228. doi: 10.1371/journal.pone.0231228 (PMC7141689; doi:10.1371/journal.pone.0231228)
Supplement: S2 Table — (DOCX) [file pone.0231228.s002.docx]

**S2 Table.** 95% Confidence Intervals by indicator: Thembisa Model

|  | Total Population | | Number of live births | | Number of live births to HIV-positive women | | HIV prevalence in females 15-49 years | | HIV prevalence in females 15-24 years | | HIV prevalence in pregnant females | |
| --- | --- | --- | --- | --- | --- | --- | --- | --- | --- | --- | --- | --- |
| Province | LL | UL | LL | UL | LL | UL | LL | UL | LL | UL | LL | UL |
| EC | 6 480 370 | 6 712 180 | 124 781 | 127 933 | 25 712 | 29 088 | 23,8% | 27,1% | 9,3% | 12,0% | 20,6% | 23,8% |
| FS | 2 838 470 | 2 922 610 | 57 221 | 582 44 | 12 702 | 14 289 | 26,0% | 29,2% | 9,9% | 12,6% | 22,2% | 25,2% |
| GP | 14 754 600 | 15 161 000 | 278 769 | 282 459 | 50 225 | 59 785 | 21,8% | 24,8% | 8,1% | 11,2% | 17,9% | 21,5% |
| KZN | 11 088 400 | 11 401 000 | 229 482 | 233 967 | 66 499 | 73 702 | 33,2% | 36,7% | 13,5% | 17,0% | 28,9% | 32,4% |
| LP | 5 717 500 | 5 880 160 | 138 928 | 141 966 | 17 473 | 22 264 | 16,3% | 20,3% | 4,6% | 7,3% | 12,6% | 16,4% |
| MP | 4 598 630 | 4 720 100 | 94 039 | 95 800 | 22 862 | 26 608 | 27,8% | 31,4% | 11,3% | 15,2% | 24,4% | 28,6% |
| NC | 1 147 180 | 1 164 990 | 25 331 | 25 565 | 2 733 | 3 292 | 13,1% | 15,3% | 4,4% | 6,1% | 10,9% | 13,2% |
| NW | 3 864 190 | 3 978 380 | 76 049 | 77 400 | 16 080 | 18 139 | 24,6% | 27,5% | 10,6% | 13,2% | 21,1% | 24,1% |
| WC | 6 742 130 | 6 867 980 | 121 150 | 122 736 | 11 184 | 17 313 | 10,4% | 16,0% | 3,6% | 6,9% | 9,2% | 14,4% |
| **RSA** | **57 546 600** | **58 766 100** | **1 158 300** | **1 173 860** | **239 664** | **262 159** | **23,8%** | **25,7%** | **9,9%** | **12,1%** | **20,7%** | **22,8%** |

**Abbreviations**: EC Eastern Cape; FS Free State; GP Gauteng Province; KZN KwaZulu-Natal; LP Limpopo Province; MP Mpumalanga Province; NC Northern Cape; NW North West; WC Western Cape; RSA South Africa; LL lower limit; UP Upper limit.
